# Ultrafast optical and passive acoustic mapping characterization of nanoscale cavitation nuclei based on gas vesicle proteins

**DOI:** 10.1063/5.0239607

**Published:** 2025-02-07

**Authors:** Cameron A. B. Smith, Avinoam Bar-Zion, Qiang Wu, Dina Malounda, Luca Bau, Eleanor Stride, Mikhail G. Shapiro, Constantin C. Coussios

**Affiliations:** 1 Division of Chemistry and Chemical Engineering, California Institute of Technology, Pasadena, California 91125, USA; 2 Department of Engineering Science, University of Oxford, Oxford OX1 3PJ, United Kingdom; 3 Andrew and Peggy Cherng Department of Medical Engineering, California Institute of Technology, Pasadena, California 91125, USA; 4 Howard Hughes Medical Institute, California Institute of Technology, Pasadena, California 91125, USA

## Abstract

Genetically encodable gas-filled particles, known as gas vesicles (GVs), have shown promise as a biomolecular contrast agent for ultrasound imaging and have the potential to be used as cavitation nuclei for ultrasound therapy. In this study, we used passive acoustic mapping techniques to characterize GV-seeded cavitation, utilizing 0.5 and 1.6 MHz ultrasound insonation over peak rarefactional pressures ranging from 100 to 2200 kPa. We found that GVs produce cavitation for the duration of the first applied pulse, up to at least 5000 cycles, but that bubble activity diminishes rapidly over subsequent pulses. At 0.5 MHz, the frequency content of cavitation emissions was predominantly broadband in nature, while at 1.6 MHz, narrowband content at harmonics of the main excitation frequency dominated. Simulations and high-speed camera imaging suggest that the received cavitation emissions come not from individual GVs but instead from the coalescence of GV-released gas into larger bubbles during the applied ultrasound pulse. These results will aid the future development of GVs as cavitation nuclei in ultrasound therapy.

Ultrasound-induced cavitation has been shown to have great potential as a therapeutic tool in a wide range of applications. Cavitation is effective in temporarily permeabilizing the blood–brain barrier,^1–3^ in transdermal delivery,^4^ and introducing material into individual cells.^5–7^ Cavitation has also shown to be a useful means of accelerating heating in thermal ablation therapies,^8–10^ while simultaneously causing direct mechanical damage to target cells.^11,12^ For example, this mechanical damage is capable both of killing tumor cells and of stimulating the immune system, leading to improved survival outcomes as well as beneficial abscopal effects.^13^

Exogenous nuclei can be utilized as a method of reducing the ultrasound energy required to generate significant levels of cavitation for use in therapies. A wide variety of particles has been explored as nuclei. For example, coated gas microbubbles have been extensively utilized as contrast agents in ultrasound diagnostic imaging, have been clinically approved for echocardiography^14^ and microvasculature Doppler imaging,^15^ and have more recently shown promise as cavitation agents for use in blood–brain barrier opening^1–3^ and cancer therapy.^16^ Solid gas-stabilizing particles^17,18^ have shown promise in enabling persistent cavitation over tens of minutes both within vascular and extravascular spaces.^11,12^

Gas vesicles (GVs) are protein-shelled gas-filled particles^19^ that can be expressed by genetically engineered bacteria^20^ and mammalian^21–23^ cells. GVs have previously been characterized for high-frequency short pulses for ultrasound imaging.^20,24–26^ Amplitude modulation imaging^27^ capitalizes on the buckling properties of GVs to generate nonlinear signals that can be exploited to achieve an improved signal-to-background ratio.^28,29^ BURST imaging^30^ takes advantage of the collapsibility of GVs with higher pressure pulses by unmixing the unique transient signal associated with GV collapse from the linear scattering background, resulting in single-cell sensitivity.

GV-producing cells are capable of infiltrating the cores of tumors^22,31^ and are capable of being engineered so that they only express the GVs under certain conditions.^30,32^ This allows for a high degree of molecular specificity, as well as enabling the GVs to populate regions of tumors that are traditionally the hardest to reach with conventional cavitation nuclei.^22,31^ Therefore, GVs occupy an interesting potential niche as a cavitation nucleation tool for ultrasound therapy. An initial study exploring their use as nucleation points for mechanical ablation has shown promise.^31^ In this work, we conduct a thorough characterization of the cavitation dynamics of GVs to guide future biomolecular and ultrasound parameters for GV-enabled cavitation-based therapy.

To characterize the GVs, we used passive acoustic mapping (PAM)—a source coherence passive beamforming process^33^ that can be implemented in the time or frequency domain.^34^ PAM captures the cavitation emissions emanating from a specific location with high SNR. We used recently developed robust Capon beamforming (RCB) PAM, which utilizes optimized beamformer weightings to reduce artifacts, further improving the SNR.^35^

The experimental setup (Fig. 1) we used to characterize the GVs comprised a single spherically focused therapeutic transducer of 64 mm active diameter and focal length 62.6 mm (H-107 D SN12; Sonic Concepts, Bothell, WA, USA), generating ultrasound at a center frequency of either 0.5 or 1.6 MHz and peak rarefactional pressures ranging from 0 to 2.2 MPa. This parameter range was chosen as it covers a wide degree of parameters that have been historically used in ultrasound therapy,^36–38^ although it does not cover the parameter range that is commonly used for histotripsy therapies. Samples were suspended in 0.5 ml volumes in 2 ml microcentrifuge tubes (Eppendorf LoBind, 2 ml; Sigma-Aldrich, Hamburg, Germany). The therapeutic transducer had a central rectangular cutout of dimensions 45–18 mm to enable the positioning of a co-axial linear array for passive acoustic mapping of cavitation. Cavitation signals generated were received using two co-planar L7-4 linear arrays (L7-4, Verasonics, Kirkland, WA, USA), one positioned inside and coaxially with the therapeutic transducer and the other at 90° to the therapeutic transducer acoustic axis, which were calibrated using a wire scatter.^39^ These signals were recorded using a programmable ultrasound engine (Vantage 256, Verasonics) and then beamformed using the RCB-PAM algorithm in a 10 mm lateral by 15 mm axial region of interest, with a pixel size of 0.4 mm square, which was chosen to be below the expected imaging resolution,^35,40^ ε = 5, as determined by visual inspection and containment of the cavitation energy to within the microcentrifuge tube. GVs were isolated from *Anabaena flos-aquae* as described previously.^41^

**FIG. 1.**
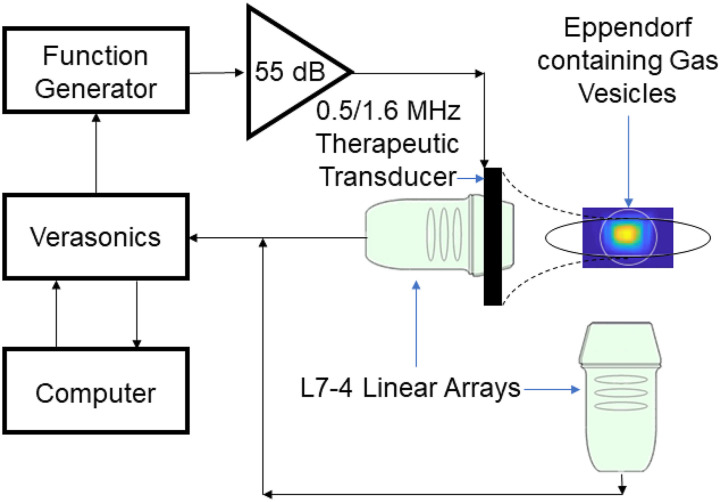
Experimental setup consisting of a therapeutic transducer transmitting either its central frequency 0.5 MHz or at the third harmonic 1.6 MHz at a range of ultrasound parameters, focused on a microcentrifuge tube containing *Anabaena* gas vesicles of a range of concentrations. The cavitation emissions are then received using two perpendicular co-planar L7-4 linear arrays for later post-processing.

First, we explored the persistence of the generated cavitation. We discovered that cavitation emissions can be detected for the duration of the applied pulse from 10 cycles up to 5000 cycles [Figs. 2(a) and 2(b)] with a pulse repetition frequency (PRF) of 0.25 Hz. However, upon subsequent pulses, the cavitation levels are greatly diminished, with very little cavitation generated after four pulses [Figs. 2(c) and 2(d)]. We also found this to be true at lower pressures, down to 200 kPa peak negative pressure. This suggests that if the intention is to maximize cavitation activity, one should focus on long pulses with a PRF set to allow for recirculation of GVs in the acoustic focus, assuming that recirculation is feasible for the required application. Due to this low persistence for all further cavitation analysis in this characterization study, we utilized only the signals emitted during the first cavitation pulse.

**FIG. 2.**
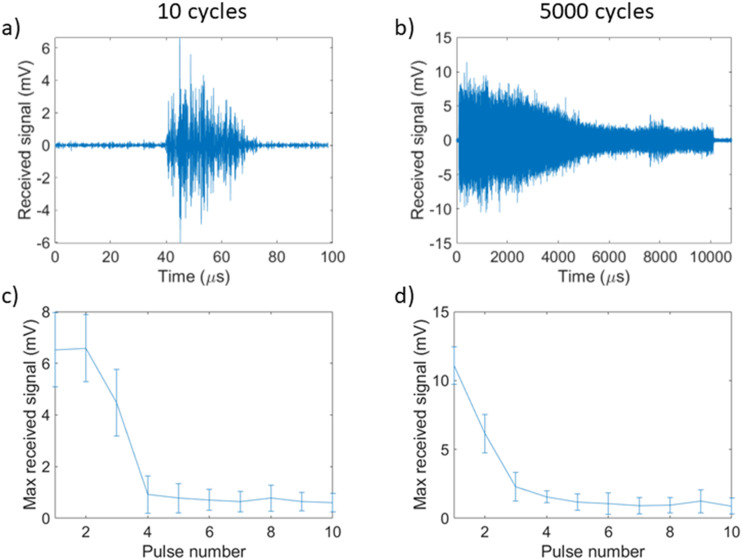
Representative example of the persistence of cavitation throughout the first transmitted pulse for (a) 10 and (b) 5000 cycles with Time = 0 being defined as the start of recording, 0.5 MHz, 1 MPa, 8 × 10^10^ particles/ml, and 0.25 Hz PRF. Maximum received signal over subsequent therapeutic ultrasound pulses averaged over ten samples with error bars representing the standard deviation for (c) 10 and (d) 5000 cycles.

Next, we examined the influence of applied pressure, both on GVs and phosphate buffered saline (PBS) control samples. Cavitation emissions from the GVs were significantly higher than from PBS, with emission levels increasing with increasing pressure as expected for both 0.5 and 1.6 MHz (Fig. 3), consistent with previous results.^31^ We separated the cavitation emissions into their broadband and harmonic components using a perfect comb filter in the frequency domain, with the filter multiplying the power spectral density of emissions by 0 at the harmonic and ultra-harmonic frequencies ±25 kHz, and by 1 everywhere else, resulting in the removal of harmonics and any broadband emissions that happened to overlap with the 50 kHz bands of the comb filter. The broadband energy lost by the filtered-out broadband signal was recovered by scaling the result by the number of 1’s and 0’s in the filter divided by the number of 1’s in the filter, with the length of the filter being determined by the useable bandwidth of the transducer. With the full energy of the broadband emissions recovered, the total energy of harmonic emissions was then estimated by subtracting the broadband energy from the energy of the total emission. As can be seen in Fig. 3, both the broadband and harmonic energies grew with increasing applied pressure, with the majority of the emissions being broadband in the case of 0.5 MHz and harmonic in the case of 1.6 MHz, even at comparable cavitation indices.^42^ The total emissions are four times stronger in the 0.5 MHz case than the 1.6 MHz case, with this being driven almost entirely by the difference in broadband emissions.

**FIG. 3.**
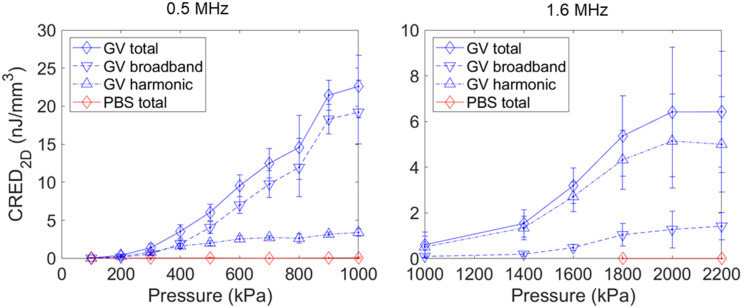
Average cavitation radiated energy density (CRED) (n = 10) split into total, broadband, and harmonic emissions over a range of applied peak negative pressures at 0.5 and 1.6 MHz incident frequency, 1.4 × 10^10^ particles/ml, with error bars representing standard deviation.

To gain a better understanding of the GV behavior during the applied therapeutic pulse, we generated spectrograms to see the distribution of emissions throughout the pulse, which we repeated at a range of particle concentrations (Fig. 4). We generated these spectrograms by averaging the spectrograms of the beamformed signals at the center of the microcentrifuge tube over ten samples to improve the SNR. In the case of the 500 kHz-stimulated emissions, some interesting temporal dynamics could be observed. Initially, a quick build-up can be observed as the harmonics and then the broadband emissions rise, until after a certain point the broadband starts to reduce and only the harmonics remain, which slowly decrease in intensity until the end of the applied acoustic pulse. The broadband signal appears to continue for a longer period at higher GV concentrations. In the case of the 1.6 MHz-stimulated emissions, consistent harmonic and ultra-harmonic emissions can be seen at all tested concentrations.

**FIG. 4.**
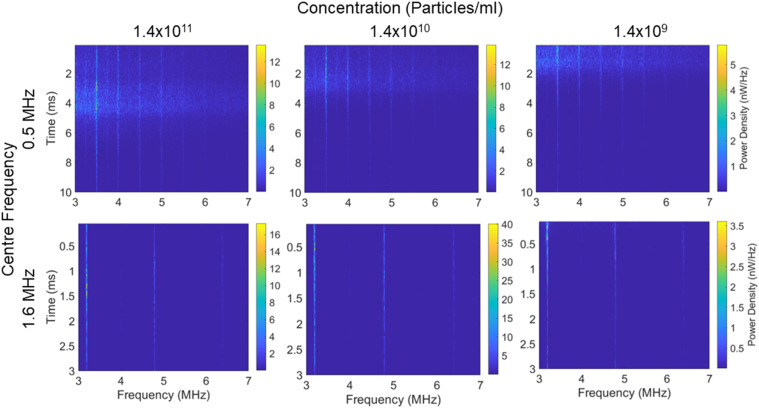
Average spectrograms over five samples showing the frequency distribution of cavitation emissions over the pulse duration for a range of particle concentrations and incident frequencies when exposed to a 5000-cycle pulse of 1 MPa in the case of 0.5 MHz and 2 MPa in the case of 1.6 MHz. Time = 0 corresponds to the time point at which cavitation emissions are expected to be received from the center of the microcentrifuge tube.

To better understand these emissions, we conducted a series of bubble simulations. Previously, it has been theorized that, under high-pressure pulses, a GV is quickly destroyed, releasing a free bubble of the gas trapped inside,^30,31^ with the subsequent radiated acoustic emissions being those that are generated from the free bubble. We used a Keller–Miksis model, as it has been previously shown to accurately predict both broadband and harmonic acoustic emissions that are in both qualitative and quantitative agreement with experimental measurements.^43^ Our parameters can be seen in Table I. We used a range of R0 values to explore bubbles with different rest sizes [Fig. 5(b)]. Of particular interest is the initial stages of insonation, Fig. 5(a) shows the experimental spectrogram from Fig. 4(b) cropped to focus on the first 100 *μ*s, with t = 0 defined as the timepoint accounting for the time of flight from the therapeutic transducer to the center of the microcentrifuge tube and back to the linear array, corresponding to the time at which one would expect to receive the start of the cavitation signal. It can be seen here that there appears to be a small delay before we begin to see the cavitation signal, the harmonic components come in, and the broadband component also grows over time.

**TABLE I.** Keller–Miksis parameters.

Parameter	Value
F	0.5 MHz
P_neg_	1 MPa
μ	1 × 10^−3^ Pa s
σ	72.5 mN/m
ρ	997 kg/m^3^
κ	1.33
c	1520 m/s

**FIG. 5.**
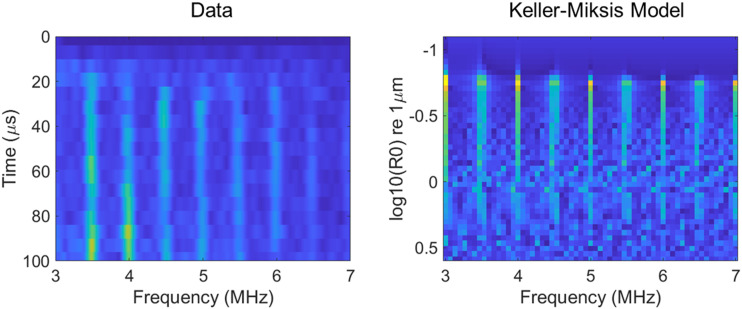
Average spectrogram (n = 5) showing the frequency distribution of cavitation emissions over the pulse duration for 0.5 MHz and 1.4 × 10^10^ particle concentration, cropped to more clearly show the cavitation emissions during the first 50 transmitted cycles. Time = 0 corresponds to the time point at which cavitation emissions are expected to be received from the center of the microcentrifuge tube (left). On the right, the results for a Keller–Miksis bubble simulation show the frequency distribution of cavitation emissions from single bubbles at a range of bubble resting sizes (R0) when exposed to the same incident ultrasound parameters and receiving array bandwidth.

A single *Anabaena* GV has an ∼85 nm diameter cylinder with a length in the range of 200–800 nm (average 519 nm).^41,44^ A sphere of equivalent volume would have a radius of roughly 90 nm. In practice, the radius of the equivalent bubble would likely be smaller than this, given that the surface tension experienced by the gas will be greater without the presence of the protein shell; however, we shall take 90 nm as a conservative estimate. It can be seen from the Keller–Miksis simulation that a bubble of this size does not give significant cavitation emissions when exposed to the ultrasound parameters used in this study; however, a larger bubble would. Therefore, we propose that the bubble emissions we record are not due to single GVs but instead originate from larger bubbles generated by the coalescence of the bubbles released from GVs. We hypothesize that the initial ultrasound cycles collapse the protein shells, releasing small gas bubbles without measurable cavitation emissions, and over the first 15 *μ*s, this gas coalesces into larger bubbles that are capable of producing harmonic and broadband emissions under continued acoustic excitation. At later time points seen in Fig. 4, the less stable broadband cavitation signal emitting bubbles eventually dissipate, forming larger, stable bubbles that emit predominantly harmonic emissions that gradually become smaller as gas diffuses slowly into the surrounding medium. The higher the total gas volume, the longer time it takes for this to occur, which explains why the period over which broadband emissions are emitted is longer at the higher GV concentrations in Figs. 4(a)–4(c).

To explore this theory, we further modified the setup (Fig. 6) to allow for the addition of a high-speed camera (HPV-X2, Shimadzu, Japan) running at 1 × 10^6^ frames per second, thereby allowing for simultaneous optical and acoustic imaging. A range of ultrasound parameters and particle concentrations were used around concentrations that have been used previously.^31^ 50-cycle pulses were used so that the entirety of the cavitation time could be recorded optically and acoustically. It is worth noting that this new setup has a significantly lower volume of sample within the focal region, with a polyethylene tube of 180 *μ*m inner diameter and 10 *μ*m wall thickness (Advanced Polymers, Salem NH, USA) being targeted instead of a 0.5 ml volume in a microcentrifuge tube. Within this new dataset, a tight threshold for cavitation emissions can be seen as demonstrated in Fig. 7, where the percentage of repeat samples (n = 30), which had at any point in time an SNR >10, is shown for a range of free-field pressures and concentrations. While using a concentration of 1.2 × 10^10^ particles per mL at a pressure of 1 MPa 90% of the samples gave cavitation emissions, if the pressure was reduced to 0.8 MPa or if the particle concentration was reduced by a factor of 1.5, then none of the 30 samples produced cavitation emissions upon exposure. Note that, at this concentration (8 × 10^9^ particles per mL), the average distance between particles is ∼5 *μ*m, with hundreds of thousands of GVs within the acoustic field of view. If we look at our optical field of view [Fig. 8(a)], with t = 0 being the frame immediately before the ultrasound arrives in the field of view, we can see that the background within the tube becomes brighter over the first 4 *μ*s, then a few bubbles, which are large enough to be viewed optically, appear, coalesce, and oscillate for the duration of 100 *μ*s long pulse, and then they dissipate upon the conclusion of the pulse (supplementary material video 1), with the background remaining at the brighter level. Figure 8(b) shows a plot of the percentage change in the background intensity across a region away from oscillating bubbles. We theorize that this background intensity corresponds to light scattering by the GVs, that is, what we observe optically is the quick destruction of all the GVs in the focal region, releasing the gas within, and this gas then either quickly dissolves into the surrounding media or, if there happens to be within the region enough gas to quickly coalesce, it does so. The gas quickly coalesces into a micrometer-sized bubble to oscillate and release the recorded acoustic emissions. This would explain the sharp threshold we see, the delay in received emissions, and how the change in optical intensity between the start and the end scales with concentration.

**FIG. 6.**
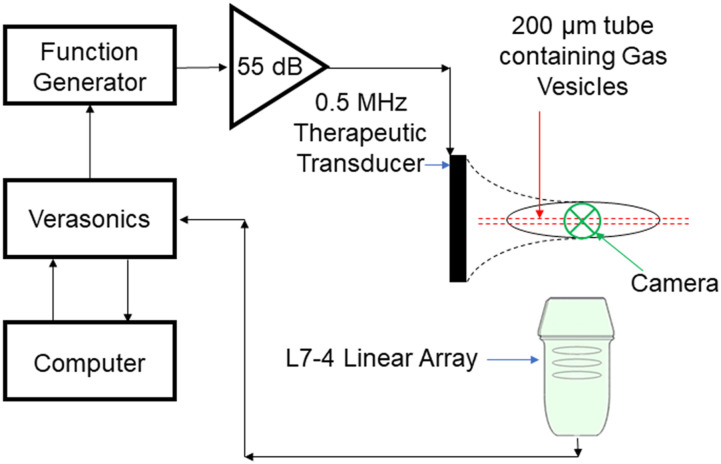
Experimental setup consisting of a therapeutic transducer transmitting its central frequency 0.5 MHz at a range of ultrasound parameters, focused on a 200 *μ*m diameter polyethylene tube containing gas vesicles at a range of concentrations. The cavitation emissions are then received using an L7-4 linear array for later post-processing.

**FIG. 7.**
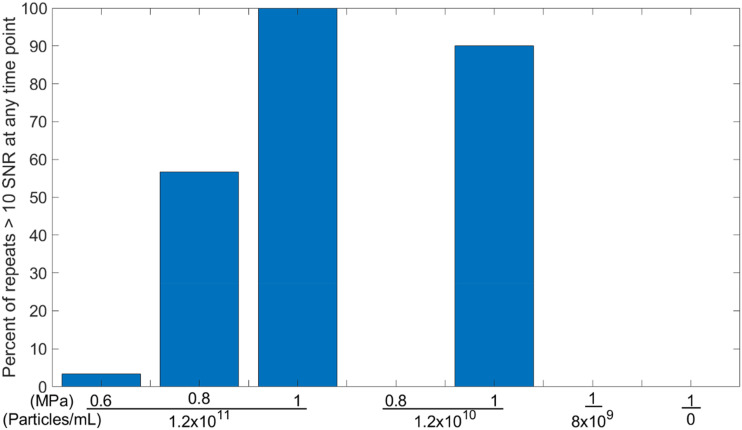
Percentage of samples (n = 30) that had at any timepoint a notable cavitation signal (defined as SNR >10) at a range of peak negative pressures and particle concentrations.

**FIG. 8.**
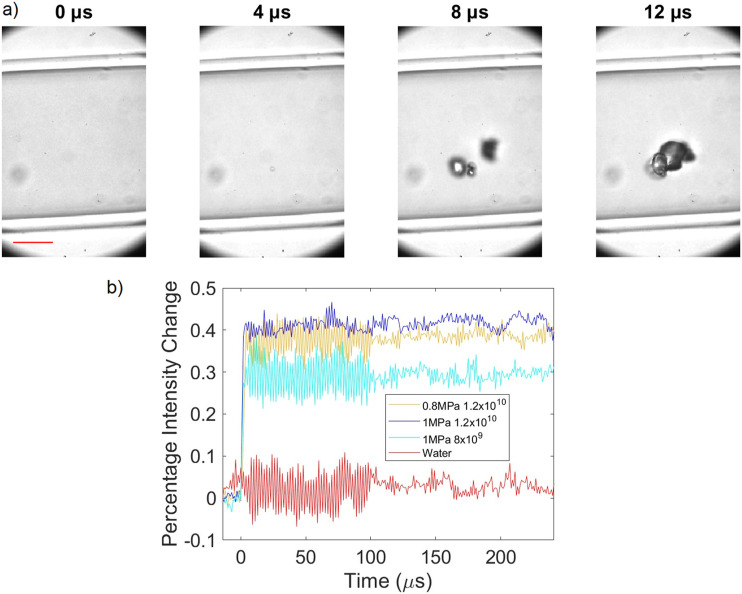
(a) Frames from a representative high-speed video at 0.8 MPa and 1.2 × 10^11^ particles/ml with Time = 0 corresponding to the time at which the incident therapeutic pulse reaches the center of the tube, scale bar = 50 *μ*m. (b) Percentage intensity change over time in the optical image in a region of interest outside of the bubbles but within the tube at a range of applied pressures and particle concentrations.

In conclusion, GVs are capable of acting as cavitation nuclei while using therapeutic pulses, although their irreversible collapse and gas release require the use of a lesser number of longer pulses to maximize the energy output. Throughout the range of pressures investigated, GVs predominantly emitted broadband cavitation signals when exposed to 0.5 MHz ultrasound and harmonic cavitation signals at 1.6 MHz. From the combined simulations and experimental evidence, it appears that the cavitation emissions detected come not from individual GVs but instead from micrometer-scaled bubbles formed by the coalescence of gas released from destroyed GVs during the emission pulse. A sharp cavitation threshold was discovered at low sample volumes both in terms of applied acoustic pressure and GV concentration, which gives further credence to the hypothesis that nanobubble release and coalescence is a necessary pre-condition to the formation of acoustically excitable cavities at therapeutic ultrasound frequencies and pressures. These results will aid in the future use of GVs as cavitation nuclei for ultrasound therapy by informing the acoustic parameters driving desired cavitation behavior, the interpretation of mechanical effects caused by GV-seeded cavitation, and the engineering of GVs and GV-expressing cells to achieve desired cavitation profiles.

See the supplementary material for a representative high-speed video at 0.8 MPa and 1.2×10^11^ particles/ml.

## Data Availability

The data that support the findings of this study are available from the corresponding author upon reasonable request.
